# Innate immune cell activation after HIV-1 vaccine administration is associated with increased antibody production

**DOI:** 10.3389/fimmu.2024.1339727

**Published:** 2024-02-13

**Authors:** Kombo F. N’guessan, Kawthar Machmach, Isabella Swafford, Margaret C. Costanzo, Lindsay Wieczorek, Dohoon Kim, Siriwat Akapirat, Victoria R. Polonis, Punnee Pitisuttithum, Sorachai Nitayaphan, Sanjay Gurunathan, Faruk Sinangil, Suwat Chariyalertsak, Julie A. Ake, Robert J. O’connell, Sandhya Vasan, Dominic Paquin-Proulx

**Affiliations:** ^1^ United States Military HIV Research Program, Walter Reed Army Institute of Research, Silver Spring, MD, United States; ^2^ Military HIV Research Program (MHRP), Henry M. Jackson Foundation for the Advancement of Military Medicine, Bethesda, MD, United States; ^3^ Military HIV Research Program (MHRP), Armed Forces Research Institute for Medical Sciences, Bangkok, Thailand; ^4^ Clinical Tropical Medicine, Mahidol University, Bangkok, Thailand; ^5^ Sanofi Pasteur, Swiftwater, PA, United States; ^6^ Global Solutions for Infectious Diseases, Lafayette, CA, United States; ^7^ Research Institute for Health Sciences, Chiang Mai University, Chiang Mai, Thailand; ^8^ Faculty of Public Health, Chiang Mai University, Chiang Mai, Thailand

**Keywords:** MAIT (mucosal-associated invariant T) cell, iNKT cell, NK cell, gamma delta (γδ) T cells, HIV vaccine, immune activation, monocytes

## Abstract

The RV144 Thai phase III clinical trial’s canarypox–protein HIV vaccine regimen showed modest efficacy in reducing infection. We therefore sought to determine the effects of vaccine administration on innate cell activation and subsequent associations with vaccine-induced immune responses. RV306 was a randomized, double-blind clinical trial in HIV-uninfected Thai adults that tested delayed boosting following the RV144 regimen. PBMC collected from RV306 participants prior to and 3 days after the last boost were used to investigate innate immune cell activation. Our analysis showed an increase in CD38+ mucosal associated invariant T (MAIT) cells, CD38+ invariant natural killer T (iNKT) cells, CD38+ γδ T cells, CD38+, CD69+ and HLA-DR+ NK cells 3 days after vaccine administration. An increase in CD14-CD16+ non-classical monocytes and CD14+CD16+ intermediate monocytes accompanied by a decrease in CD14+CD16- classical monocytes was also associated with vaccine administration. Inclusion of ALVAC-HIV in the boost did not further increase MAIT, iNKT, γδ T, and NK cell activation or increase the proportion of non-classical monocytes. Additionally, NK cell activation 3 days after vaccination was positively associated with antibody titers of HIV Env-specific total IgG and IgG1. Vδ1 T cell activation 3 days after vaccine administration was associated with HIV Env-specific IgG3 titers. Finally, we observed trending associations between MAIT cell activation and Env-specific IgG3 titers and between NK cell activation and TH023 pseudovirus neutralization titers. Our study identifies a potential role for innate cells, specifically NK, MAIT, and γδ T cells, in promoting antibody responses following HIV-1 vaccine administration.

## Introduction

The RV144 HIV vaccine regimen consisting of ALVAC-HIV, an attenuated non-replicating canarypox virus vector given at weeks 0, 4, 12, and 24, and AIDSVAX B/E, a bivalent recombinant gp120 protein derived from HIV-1 CRF01_AE and B subtypes given at weeks 12 and 24, demonstrated a modest efficacy of 31.2% in preventing HIV acquisition in a phase III clinical trial in Thailand (1). Subsequent studies identified an inverse correlation between binding of IgG antibodies to variable regions 1 and 2 (V1V2) of HIV-1 envelope proteins (Env) and the rate of HIV-1 acquisition as well as a direct correlation between binding of plasma IgA antibodies to Env and the rate of acquisition (2, 3). In addition, IgG3 antibodies correlated with a decreased risk of infection (3). Furthermore, CD4 T cell functionality and polyfunctionality scores, as calculated by COMPASS, correlated inversely with HIV acquisition (4). RV306 was a follow up phase I trial also conducted in Thailand that administered the RV144 regimen with an additional boost consisting of AIDSVAX B/E gp120 alone or together with ALVAC-HIV (5). The additional boost increased the magnitude and durability of CD4 T cell responses, binding antibodies, as well as pseudovirus neutralization and there was no difference in the primary analysis between the two boosting strategies. The innate immune response to the first ALVAC-HIV administration has been described using samples from a phase 1b trial of the RV144 regimen in HIV-1 uninfected South Africans (HVTN 097) (6). The study found that Type I and II interferon signaling pathways and innate pathways critical for adaptive immune priming were activated 1 day post vaccination. However, the involvement of innate cells’ sensing of the vaccine components after subsequent vaccinations remains to be elucidated.

Innate cells play a critical role in shaping adaptive immune responses through the production of immunoregulatory cytokines and through direct-cellular interactions in the case of NK cells and antigen presenting cells (7–11). Peripheral blood NK cells are mostly mature and cytotoxic and are identified as CD56^dim^ NK cells (12). A smaller proportion of NK cells known as immature or early CD56^hi^ can produce IFNγ upon stimulation by cytokines (12). NK cells have been identified as a major source of IFNγ following vaccination with ALVAC-HIV (13). Additionally, studies in a non-human primate (NHP) model indicated that NK cell-induced activation of monocytes following vaccination with ALVAC-SIV decreased risk of SIVmac251 acquisition (14). Recent studies have identified a monocyte-derived gene signature associated with reduced risk of HIV acquisition in RV144 (11). Circulating monocytes can be divided into distinct subtypes based on CD14 and CD16 expression. The classical monocytes, defined as CD14+CD16-, comprise the majority of monocytes in peripheral blood. Furthermore, studies in NHP have shown that classical monocytes are a strong correlate of decreased risk of SIV acquisition by an RV144 like vaccine regimen (14).

Unconventional T cells include MR1-restricted mucosal-associated invariant T cells (MAIT cells), CD1d-restricted natural killer T cells (NKT cells), and gamma delta (γδ) T cells. MAIT cell have been recently shown to be able to provide B cell help (15, 16) and promote the maturation of dendritic cells (17, 18) via CD40L as well as via the production of cytokines such as IL-21. MAIT cells are activated by vaccination with the ChAdOx01 viral vector, and their activation was essential for induction of antigen specific CD8 T cells (19). Similarly, MAIT cell characteristics have been associated with the adaptive immune response following mRNA vaccination (9, 20). Invariant NKT (iNKT) cells can quickly produce cytokines capable of activating several immune cells, including NK cells, dendritic cells, other unconventional T cells, and B cells (21). These attributes of iNKT cells make them potential immunoregulators in the context of vaccines. Studies have shown that co-injection of iNKT cell agonists, together with antigens promotes humoral and cellular immune responses (22–25). γδ T cells represent a subset of unconventional T cells defined by the expression of T-cell receptors (TCRs) composed of γ and δ chains, differentiating them from classical CD4+ and CD8+ T cells which express αβ TCRs (26). γδ T cells are capable of recognizing markers of cellular stress resulting from infection and display a broad function through the production of cytokines such as IFNγ, TNF-α and IL-17, chemokines such as RANTES and IP-10 and cytolytic proteins such as perforin and granzyme B (27). In humans, γδ T cells can be grouped into two broad subsets characterized by their TCR δ chain usage: Vδ1 T cells are predominantly found in the thymus and peripheral tissues while Vδ2 T cells are the main subset present in blood (27). γδ T cells are capable of regulating B cells by influencing B cell differentiation and promoting CD4 T cells secretion of IL-13 and IL-21 (8, 26, 28). Furthermore, studies in human peripheral blood have identified a subset of Vγ9/Vδ2 + γδ T cells expressing CXCR5 which can be induced to express costimulatory molecules ICOS and CD40L, secrete IL-2, IL-4, and IL-10, and help B cells in antibody production (29).

Understanding innate immune cell responses to the vaccination could provide us with valuable insights on strategies to improve HIV-1 vaccine regimens. Here, we describe innate immune cell responses in the context of delayed boosting of the RV144 regimen with ALVAC-HIV and AIDSVAX B/E in RV306 (5). In our study, we observe increased NK, MAIT, and γδ T cell activation as well as an increase in CD14+CD16+ monocytes 3 days following late-stage boost vaccination. Importantly, NK cells, MAIT cells, and Vδ1 T cells activation was associated with the promotion of antibody responses while CD14+CD16+ monocytes were negatively associated with CD4+ T cell functionality and polyfunctionality scores.

## Materials and methods

### Study approval

The RV306 study was approved by ethical review boards at the Walter Reed Army Institute of Research, Thai Ministry of Public Health, Royal Thai Army Medical Department, Faculty of Tropical Medicine, Mahidol University, Chiang Mai University, and Chulalongkorn University Faculty of Medicine. All study participants provided informed consent. The investigators have adhered to the policies for protection of human participants as prescribed in AR 70-25.

### Study design and participants

The RV306 clinical trial (ClinicalTrials.gov NCT01931358) was a double-blind, placebo-controlled, randomized clinical trial conducted in healthy Thai volunteers as previously described (5). Study participants included in this analysis received the RV144 vaccine regimen consisting of viral vector ALVAC-HIV prime at weeks 0 and 4 followed by ALVAC-HIV and AIDSVAX B/E protein boost at weeks 12 and 24 and received an additional boost of either ALVAC-HIV and AIDSVAX B/E (group 2) or AIDSVAX B/E alone (group 3) administered at week 48 (**Supplementary Figure 1**). Peripheral blood mononuclear cells (PBMC) collected from participants in the active arms of groups 2 (n=7) and 3 (n= 9) prior to and 3 days after the last boost were used to investigate innate immune cell activation.

### Innate immune cell staining

Cryopreserved PBMC were thawed in 5ml of thawing medium containing benzonase nuclease in DMEM + 20% FBS. Following washes, cells were stained with LIVE/DEAD Fixable Blue Dead (Thermo Fisher, Cat L34957) in PBS for 30 min at room temperature. FcR blocking was conducted for 15 min at room temperature with 10% normal mouse IgG (Thermo Fisher, Cat OB2040-09) in staining buffer (PBS containing 0.1% NaN*3* and BSA). Cell surface staining was conducted using a cocktail of fluorescently labelled antibodies (BD Biosciences unless otherwise indicated): CD69 BB660 (Custom), CD158a/h/g (KIR2DL1/S1/S3/S5) PerCP-Cy5.5 (BioLegend, Cat 339514), CD158e1 (KIR2DL1) PerCP-Cy5.5 (BioLegend, Cat 312718), CXCR5 BB790-P (Cat 755631), Vb11 APC (Beckman, Cat A66905), CD161 R718 (Cat 751652), CD8 APC Cy7 (Cat 557760), CD56 BV421 (Cat 568219), HLA-DR BV480 (Cat 566113), AQUA L/D, Va7.2 BV711 (BioLegend, Cat 351732), CD33 BV786 (Cat 740974), CD19 BV786 (Cat 563325), CD57 BUV395 (Cat 567621), CD16 BUV496 (Cat 612944), TCRd2 BUV563 (Cat 748582), PD-1 BUV661 (Cat 750260), CD38 BUV737 (Cat 612824), CD4 BUV805 (Cat 612887), Va24 PE (Beckman, Cat IM2883), NKG2C PE-Dazzle594 (Miltenyi, Cat 130-123-047), CD3 PECy5.5 (Thermo Fisher, Cat 35-0036-42), TCRd1 PE Cy7 (Thermo Fisher, Cat 25-5679-42), CD80 FITC (BioLegend, Cat 305206), CD83 APC Cy7 (BioLegend, Cat 305330), CD86 BV605 (BioLegend, Cat 305430) in staining buffer with Brilliant Stain Buffer (Thermo Fisher, Cat 00-4409-42) for 15 min at room temperature. Intracellular staining was performed after fixation and permeabilization using FIX & PERM Cell Permeabilization Kit (Invitrogen, Cat GAS003) and a cocktail of fluorescent antibodies against intracellular proteins: FcRg FITC (1:80) (Millipore, Cat FCABS400F) and Ki67 BV750 (1:80) (Custom).

### Flow cytometry

Flow cytometry data was collected on a FACSymphony A5 flow cytometer (Becton Dickenson, Franklin Lakes, NJ, USA) and analyzed using FlowJo version 10.8.1 software for Mac OS (Becton Dickenson, Franklin Lakes, NJ, USA). The gating strategy is shown in **Supplementary Figure 2**.

### Statistical analysis and correlates

Statistical analysis was performed using GraphPad Prism version 9.4.1 for Mac OS. Paired comparisons within a group were performed using a Wilcoxon test. Comparisons between groups were performed using the Mann–Whitney test. The median and SD for all group comparisons can be found in the **Supplemental Tables File**. Env TH023 specific COMPASS functionality scores and polyfunctionality scores for CD4 T cells have been previously reported (5, 30). Antibody titers against gp120 A244 and Env TH023 specific neutralization were previously measured (5, 30, 31). A spearman test was used to calculate correlations between innate cell phenotypes and effector functions, functionality scores and antibody titers. P values below 0.05 were considered significant.

### Random forest

A Random Forest algorithm (‘randomForest()’ function in R) was evaluated. We configured our settings to generate 500 trees. For each node split, the algorithm considered a subset of predictors equal to the square root of the total number of available variables. The importance of each attribute was quantified using reductions in the Gini Impurity, facilitating a hierarchical feature ranking that underscores their discriminative power in distinguishing between pre- and post-vaccination states.

## Results

### Conventional T cells and unconventional T cells are activated 3 days post vaccination with ALVAC-HIV and AIDSVAX B/E

**Figure 1:**
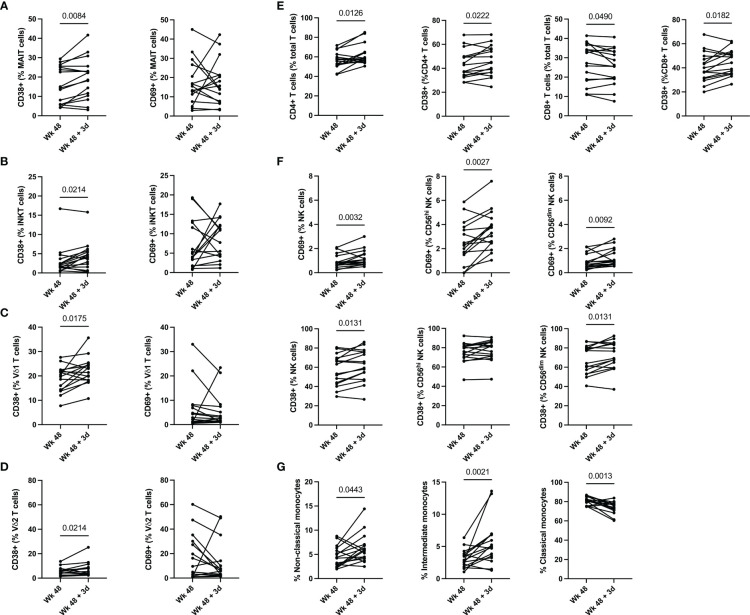
Innate and T cell activation 3 days post vaccination. **(A)** MAIT, **(B)** iNKT, **(C)** Vδ1 T, **(D)** Vδ2 T cell, **(E)** conventional T cells and **(F)** NK cell activation pre- and post-vaccination. **(G)** Changes in monocyte frequency pre- and post-vaccination. Classical monocytes are defined as CD14+CD16-, intermediate monocytes as CD14+CD16+ and non-Classical monocytes are defined as CD14-CD16+. Groups 2 (ALVAC-HIV + AIDSVAX B/E) and 3 (AIDSVAX B/E) are combined for analysis (n=16).

To elucidate the function of T cells following the administration of the ALVAC-HIV and AIDSVAX B/E vaccine regimen, we determined the frequency and phenotype of MAIT, iNKT, γδ T cells, and conventional T cells immediately prior to vaccine administration and 3 days post last vaccination in RV306 (**Supplementary Figure 1**) by flow cytometry (**Supplementary Figure 2**). There was no change in MAIT cell frequencies 3 days post vaccination (**Supplementary Figure 3**). We observed an increase in the levels of CD38 expressing MAIT cells 3 days post vaccination (p=0.0084) (**Figure 1A** and **Supplementary Figure 5**). The NK cell receptor, CD56, can be used to denote two distinct MAIT cell populations (32). MAIT cells expressing CD56 exhibit a greater ability to respond to IL-12 and IL-18 compared to CD56- MAIT cells (32). Our results show that the increase in CD38 positive MAIT cells occurred in both CD56+ MAIT cells (p=0.0088) and CD56- MAIT cells (p=0.0028) (**Supplementary Figure 4B**).

Similarly to MAIT cells, we observed an increase in CD38 positive iNKT cells 3 days post vaccination (p=0.0214) (**Figure 1B** and **Supplementary Figure 5**) and there was no change in their frequency (**Supplementary Figure 3**). Functionally, iNKT cells can be categorized into 3 subsets, CD4+ iNKT cells which produce both Th1 and Th2 cytokines, and CD8+ iNKT cells and CD4-CD8- (DN) iNKT cells which exhibit a Th1 response with a greater propensity for cytolytic activity (33). Our data shows an increase in CD8+ iNKT cells following vaccine administration (p=0.0114) (**Supplementary Figure 4A**). This increase in CD8+ iNKT cells is accompanied by a decrease in DN iNKT cells (0.0010) (**Supplementary Figure 4A**).

γδ T cell activation post vaccination was also increased, as measured by CD38 positive Vδ1 (p=0.0175) and Vδ2 T cells (p=0.0214), 3 days post vaccination with ALVAC-HIV and AIDSVAX B/E (**Figures 1C, D** and **Supplementary Figure 5**). There was no change in the frequency of Vδ1 and Vδ2 T cells 3 days following vaccination (**Supplementary Figure 3**).

We also observe an increase in frequency of CD4+ T cells (p=0.0126) and a decrease in CD8+ T cells (p=0.0182) 3 days post vaccination (**Figure 1E**). This was accompanied by an increase in CD38 expression in CD4+ T cells (p=0.0222) and CD8+ T cells (p=0.0182). There was also a trend for an increase in circulating CXCR5+ CD4 T cells post vaccination but it did not reach significance (data not shown). Overall, our results show activation of unconventional and conventional T cells, as measured by CD38 expression, 3 days post last vaccination in RV306.

### Natural killer cell are activated by ALVAC-HIV and AIDSVAX B/E vaccine regimen

Next, we investigated the frequency and activation of NK cells following ALVAC-HIV and AIDVAX B/E vaccine administration within total NK cells and the CD56^hi^ and CD56^dim^ subsets (**Figure 1F**). Overall, there was no change in the frequency of NK cells 3 days post last vaccination (**Supplementary Figure 3**). We found an increase in the levels of CD38 (p=0.0131), CD69 (p=0.0032) (**Figure 1F**) and HLA-DR (p=0.0270) in total NK cells three days post vaccination (**Figure 1F** and **Supplementary Figure 4C**). Within the CD56^hi^ NK cell compartment, we found an increase in CD69 (p=0.0027) and HLA-DR (p=0.0042) expression following vaccination (**Figure 1E** and **Supplementary Figure 4C**). Finally, we observed an increase in levels of CD38 (p=0.0131) and CD69 (p=0.0092) on CD56^dim^ NK cells following vaccination (**Figure 1F**). Natural killer cells co-expressing NKG2C and CD57 have an adaptive memory-like phenotype (34). Functionally, these adaptive memory-like NK cells have decreased cytolytic capacity and response to cytokines (IL-2 and IL-18) produced by innate cells and increased CD16-mediated antibody-dependent cellular cytotoxicity (ADCC) (34). Our study shows that this NK cell compartment is also activated following vaccination (CD38; p=0.0035) (**Supplementary Figure 4C**). These results suggest that several subsets of NK cells were activated in response to vaccination in RV306.

### Increase in CD16+ monocytes following vaccination with ALVAC-HIV and AIDSVAX B/E

Monocytes play a key role in the protection conferred through vaccination by RV144 based vaccines (11, 14, 35). We therefore measured the frequency of monocyte subsets post-vaccination with ALVAC-HIV and AIDSVAX B/E (**Figure 1G**). We found a decrease in classical CD14+CD16- monocytes 3 days following vaccination (p=0.0013), this was accompanied by an increase in intermediate CD14+CD16+ monocytes (p=0.0021) and non-classical CD14-CD16+ monocytes (p=0.0443) (**Figure 1G**). Next, we evaluated expression of costimulatory molecules, CD80, CD83 and CD86 on total monocytes and found no change in expression of any of the markers (data not shown).

### Conventional T cell, MAIT, iNKT, and γδ T cell activation three days post-vaccination does not require ALVAC-HIV

**Figure 2:**
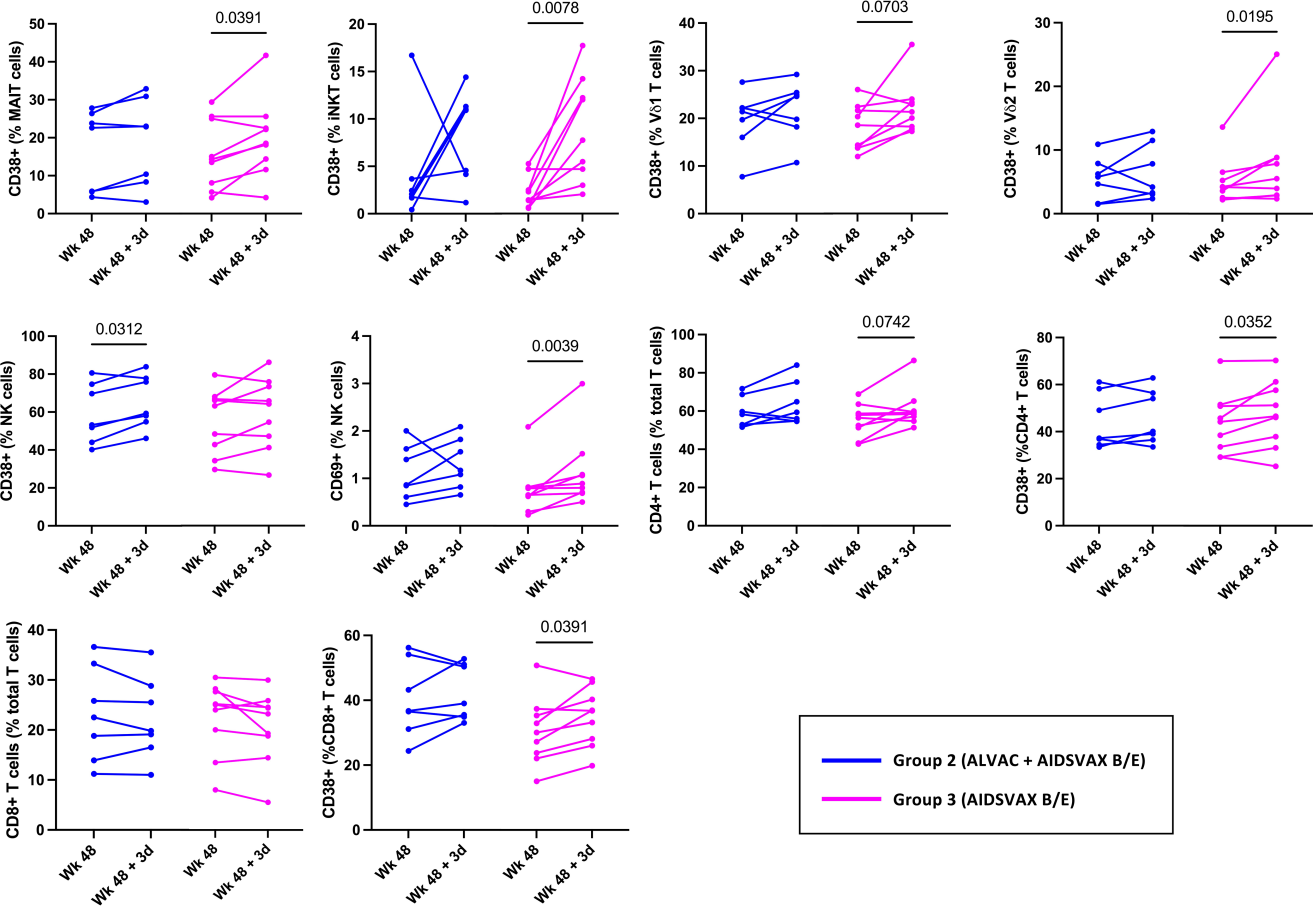
Innate and T cell activation in RV306 study participants that received ALVAC-HIV and AIDSVAX B/E or AIDSVAX B/E alone. Activation of MAIT, iNKT, NK, γδ T cells, and conventional T cell was analyzed in 7 participants from group 2 (ALVAC-HIV + AIDSVAX B/E) and 9 participants from group 3 (AIDSVAX B/E).

In RV306, the last vaccination consisted of both ALVAC-HIV and AIDSVAX B/E for group 2 while group 3 received AIDSVAX B/E alone (5). Alum was used as the adjuvant in both groups. To investigate any group specific effects of the vaccine on innate cell activation, we evaluated activation in each study group (**Figure 2**). The increase in CD38 expression by MAIT (p=0.0391), iNKT (p=0.0078), γδ T cells (Vδ1, p=0.0703; Vδ2, p=0.0195), CD4 T cells (p=0.0352), CD8 T cells (0.0391) and CD69 levels on NK cells (p=0.0039) was significant in the AIDSVAX B/E only group. Interestingly, increased expression of CD38 by NK cells was significant in the ALVAC-HIV and AIDSVAX B/E group (p=0.0312) and not in the AIDSVAX B/E only. However, the number of study participants in each group was low and a pattern of increased activation was present in both groups.

### Activation of unconventional T cells and NK cells 3 days post vaccination with ALVAC-HIV and AIDSVAX B/E is associated with humoral immune responses

**Figure 3:**
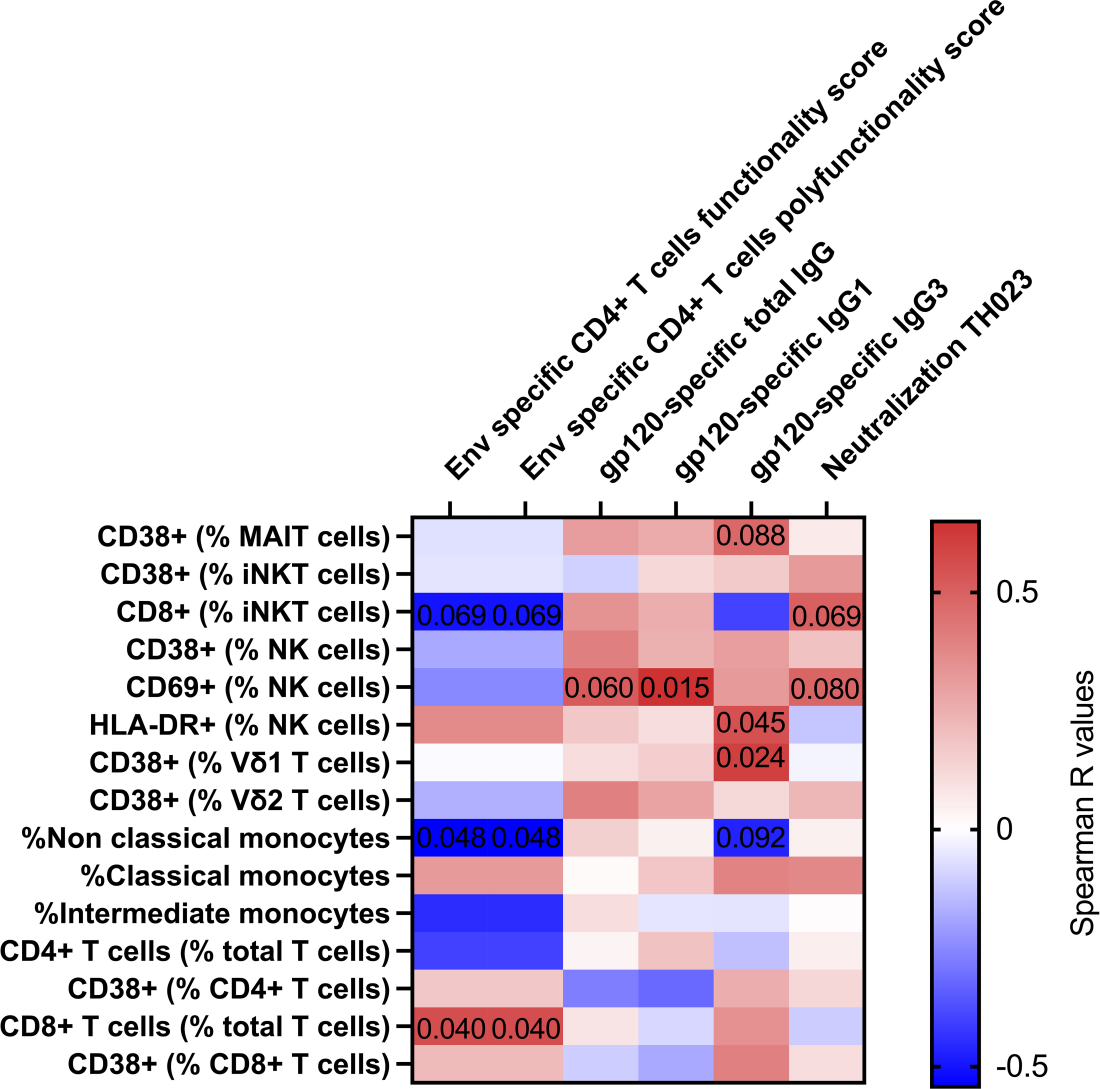
Correlation between innate and T cell activation with adaptive immune responses 14 days post-vaccination. Heat map showing the Spearman R values for associations between immune activation at day 3 (Y axis) and adaptive immune responses at day 14 post vaccination (X axis). Significant and trending p-values are denoted on the heatmap. Classical monocytes are defined as CD14+CD16-, intermediate monocytes as CD14+CD16+ and non-Classical monocytes are defined as CD14-CD16+. All participants from group 2 and 3 were included in the heatmap analysis (n=16).

Finally, we looked at associations between immune cell activation and adaptive immune responses measured 14 days post last vaccination previously reported (5) (**Figure 3**). NK cell activation 3 days after vaccine administration was associated with antibody titers of HIV gp120-specific total IgG (r=0.517; p=0.060), IgG1 (r=0.649; p=0.015) and IgG3 (r=0.548; p=0.045). Vδ1 T cell activation 3 days after vaccine administration associated with HIV gp120-specific IgG3 titers (r=0.604; p=0.024). The frequency of CD14-CD16+ monocytes was inversely associated with HIV Env specific CD4+ T cell functionality score and polyfunctionality score (r=-0.543; p=0.048). Finally, we also observed trending associations between MAIT cell activation and gp120-specific IgG3 titers (r=0.475; p=0.088), between NK cell activation and TH023 pseudovirus neutralization titers (r=0.486; p=0.088), and between CD8+ iNKT and TH023 pseudovirus neutralization titers (r=0.503; p=0.069), HIV Env specific CD4+ T cell functionality score (r=-503; p=0.069) and polyfunctionality score (r=-503; p=0.069). We also observed an association between CD8+ T cell frequency and HIV Env specific CD4+ T cell functionality score and polyfunctionality score (r=0.5598; p=0.040). Overall, this suggests that unconventional T cell and NK cell activation induced by vaccination might have contributed to the development of humoral immune responses in RV306. Lastly, using random forest analysis, we identified classical monocytes as the variable having the higest ability to discriminate between samples prior to and 3 days post the last boost (**Supplementary Figure 6**).

## Discussion

In this study, we investigated T cell and innate cells’ involvement in sensing ALVAC-HIV and AIDVAX B/E and determined associations between our findings and previously identified vaccine induced humoral and cellular responses. Using samples from RV306 which tested delayed boosting following the RV144 regimen, we report increased NK cell, MAIT cell, iNKT, γδ T, CD4+ T cell and CD8+ T cell activation 3 days following late-stage boost vaccination. We also report an increase in CD14+CD16+ monocytes following late-stage boost vaccination. Inclusion of ALVAC-HIV (group 2) was not required for innate and conventional T cell activation 3 days post vaccination. It is possible that any effect conferred by ALVAC-HIV on innate cell activation is no longer detectable after 3 days. Our analysis included additional markers such as PD-1, HLA-DR and CXCR5 in T cells and CD80, CD83 and CD86 costimulatory molecules for monocytes, however we did not see any significant changes in the expression of these markers. Although there was trending increase in circulating CXCR5+ CD4 T cells, there was no change in their expression of activation markers.

Previous work using samples at days 1, 3 and 7 post first vaccination from a phase 1b trial using the RV144 regimen in HIV-1 uninfected South Africans (HVTN097) found that transcriptional responses peaked 1 day post vaccination (6). Type I and II interferon signaling and innate pathways critical for adaptive immune priming were activated. Furthermore, this study reported that innate signatures at day 1 were positively associated with antibody-dependent cellular cytotoxicity (ADCC) and phagocytosis (ADCP) at 6.5 months (2 weeks post final vaccination). Conversely, the study found that day 3 and day 7 innate immune signatures were inversely associated with Env-specific CD4+ T cell responses at 6.5 months and 12 months (6 months post last vaccination) indicating that a quick resolution of this signature was associated with higher Env-specific CD4+ T cell responses. Similar to our findings, increased CD14+CD16+ monocytes following ALVAC-HIV administration was observed in the HVTN097 study (6).

Interestingly, NK, MAIT and Vδ1 T cell activation 3 days post vaccination was associated with the magnitude of gp120 specific IgG1 and IgG3 antibody responses and the increase in CD14+CD16+ monocytes was negatively associated with HIV-specific CD4+ T cell functionality and polyfunctionality scores. Production of gp120 specific IgG3 has been associated with a lower risk of acquisition in RV144 (3) and COMPASS CD4 T cell functionality and polyfunctionality correlated inversely with HIV acquisition (4). MAIT cells have been shown to provide B cell help (15, 16) and promote the activation of dendritic cells (17, 18). In addition, cytokines, such as IFNγ, produced by NK cells may promote B cell activation and enhance antibody production (36, 37). γδ T cells are capable of regulating adaptive immune responses (8, 26, 28). Although our study identifies trending correlations between CD8+ iNKT subset and humoral responses, previous studies by Liu et al. (38) showed little functional differences between the CD4- CD8- and CD8+ iNKT cell subsets. In addition, it is unclear whether the changes observed in the iNKT cell subsets are permanent changes or temporal. Additional longitudinal analysis is required to determine this. More work is also needed to understand how NK cell and unconventional T cells activation following vaccination could promote humoral responses. While we also observed conventional CD4 and CD8 T cells activation 3 days post vaccination, it was not associated with the magnitude of humoral immune responses, suggesting a different role for conventional and unconventional T cells.

The mechanism responsible for the innate cell activation that we reported here remains to be investigated. Interestingly, activation of both MAIT and Vδ2 T cells following vaccination with the ChAdOx01 viral vector was dependent on IL-18, TNF, and type I IFN (19, 39). It is possible that a similar pathway was activated following vaccination with ALVAC-HIV and AIDSVAX B/E. One limitation of our study is that there were no samples collected 1 day post vaccination, when innate immune responses are expected to peak. Our results show that 3 days post vaccination, CD69+ NK cells correlated with humoral responses, this was not the case for CD38+ NK cells. This may be due to differing dynamics between CD38 and CD69. As such, it is possible that CD38+ NK cells would also be associated with humoral responses if sampling had occurred at a different time point. The number of study participants was also small when dividing between those who received ALVAC-HIV and AIDSVAX B/E and those who received AIDSVAX B/E alone. Contrasting the innate immune response induced by different adjuvants would be of great interest to better understand how to fine tune adaptive immune responses. Lastly, an additional limitation to this study is that our findings are only correlative. Further investigation is still needed to corroborate the effect of innate cells in modulating adaptive immune responses to ALVAC-HIV and AIDSVAX B/E.

This study suggests that unconventional T cells and NK cell’s sensing of vaccine components could contribute to vaccine induced humoral responses. A better understanding of how different boost strategies modulate innate immune responses to adjuvants used in vaccines and modulate the elicitation of differing antibody subtypes could help improve HIV vaccine designs.

## Data availability statement

The raw data supporting the conclusions of this article will be made available by the authors, without undue reservation.

## Ethics statement

The RV306 study was approved by ethical review boards at the Walter Reed Army Institute of Research, Thai Ministry of Public Health, Royal Thai Army Medical Department, Faculty of Tropical Medicine, Mahidol University, Chiang Mai University, and Chulalongkorn University Faculty of Medicine. The studies were conducted in accordance with the local legislation and institutional requirements. The participants provided their written informed consent to participate in this study.

## Author contributions

KN: Formal Analysis, Investigation, Methodology, Writing – original draft. KM: Investigation, Methodology, Writing – original draft. IS: Investigation, Methodology, Writing – review & editing. MC: Methodology, Resources, Writing – review & editing. LW: Methodology, Resources, Writing – review & editing. DK: Writing – review & editing, Formal analysis. SA: Methodology, Resources, Writing – review & editing. VP: Methodology, Resources, Writing – review & editing. PP: Methodology, Resources, Writing – review & editing. SN: Project administration, Resources, Writing – review & editing. SG: Resources, Writing – review & editing. FS: Resources, Writing – review & editing. SC: Project administration, Resources, Writing – review & editing. JA: Project administration, Resources, Writing – review & editing. RO: Project administration, Resources, Writing – review & editing. SV: Project administration, Resources, Writing – review & editing. DP: Conceptualization, Supervision, Writing – original draft.

## Author disclaimer

Material has been reviewed by the Walter Reed Army Institute of Research. There is no objection to its presentation and/or publication. The opinions or assertions contained herein are the private views of the author and should not be construed as official, or as reflecting true views of the Department of the Army, the Department of Defense, or the Henry M. Jackson Foundation for the Advancement of Military Medicine. The investigators have adhered to the policies for protection of human subjects as prescribed in AR 70-25.
