# Supplementary material for: Innate immune cell activation after HIV-1 vaccine administration is associated with increased antibody production
Source: Front Immunol. 2024 Feb 13;15:1339727. doi: 10.3389/fimmu.2024.1339727 (PMC10900843; doi:10.3389/fimmu.2024.1339727)
Supplement: Supplementary file 1 [file DataSheet_1.pdf]

RV306 Study Group

Mahidol University: Arom Pitisuthitham, Yupa Sabmee

RTA-AFRIMS: Narongrid Sirisopana, Chirapa Eamsila, Prapaporn Savaraj, Wanlaya Labwech, Siriluck Teerachia

Research Institute for Health Sciences (RIHES), Chiang Mai University: Nuntisa Chotirosniramit, Taweewat Supindham, Boonlure Pruenglampoo, Patcharaphan Sugandhavesa, Natthapol Kosashunhanan, Oranitcha Kaewthip, Piyathida Sroysuwan, Somporn Tipsuk, Carlo Sacdalan

BIOPHICS, Mahidol University: Pawinee Jarujareet

US Military HIV Research Program: Silvia Ratto-Kim, Sebastian Molnar, Jesse Schoen, Merlin L. Robb, Michael A. Eller

USAMD-AFRIMS: Nampueng Churikanont, Saowanit Getchalarat, Nongluck Sangnoi, Bessara Nuntapinit, Anant Phramtong, Pornsuk V. Grandin, Sirinan Madnote, Surawach Rittiroongrad, Boot Kaewboon, Rapee Trichavaroj, Jiraporn Puangkaew, Somsak Chantakulkij, Phiromrat Rakyat, Pornchanok Panjapornsuk, Nipattra Tragonlugsana, Weerawan Chuenarom, Mark de Souza, Viseth Ngauy

Thai Red Cross AIDS Research Centre: Nittaya Phanuphak, Nitiya Chomchey, Puttachard Saengtawan, Nipat Teeratakulpisarn, Eugene Kroon

Faculty of Medicine, Chulalongkorn Hospital: Rungsun Rerknimitr

Global Solutions for Infectious Diseases: Carter A. Lee

United States Army Medical Materiel Development Activity (USAMMDA): Suchada Chinaworapong

International Vaccine Institute: Jerome H KIM

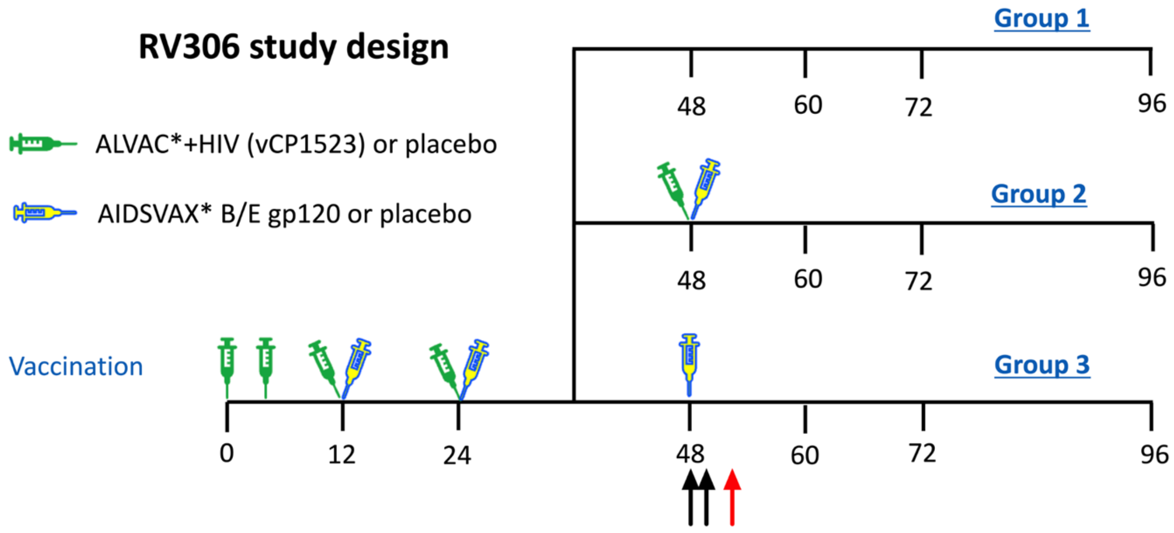

Supplemental Figure 1. RV306 study design. Study participants received the RV144 vaccine regimen and no additional boost (group 1), ALVAC-HIV and AIDSVAX B/E at week 48 (group 2), or AIDSVAX B/E alone at week 48 (group 3). Complete RV306 study design is available in Pitisuttithum et al. PBMCs from week 48 and week 48 + 3 days (black arrow) were analyzed by flow cytometry. Antibody titers and neutralization as well as Env TH023 specific CD4+ T were evaluated at week 50 [1, 2](red arrow).

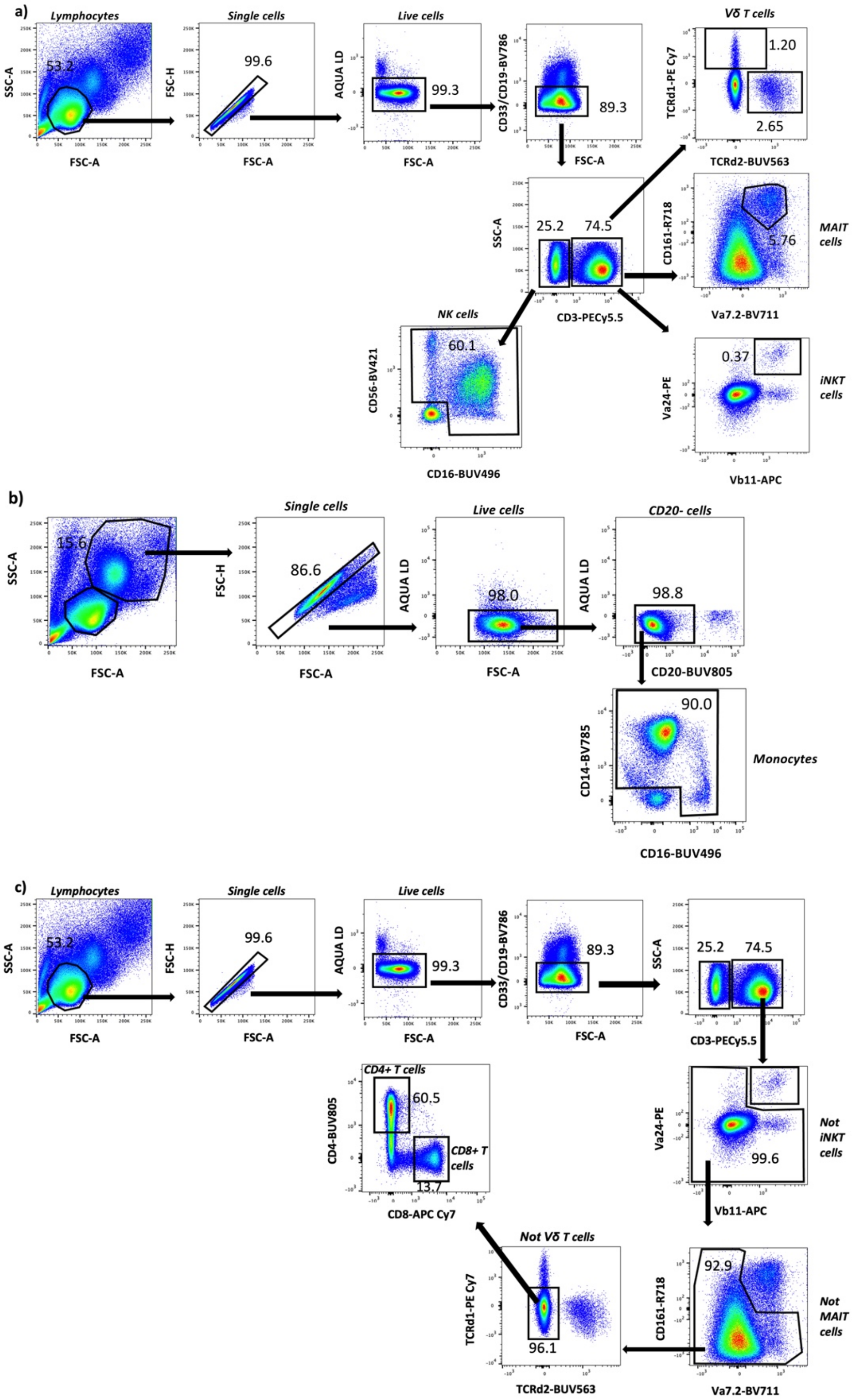

Supplemental Figure 2: Gating strategy for MAIT, iNKT, Vδ T cell, NK cell (a), monocyte b), and conventional CD4+ and CD8+ T cells (c).

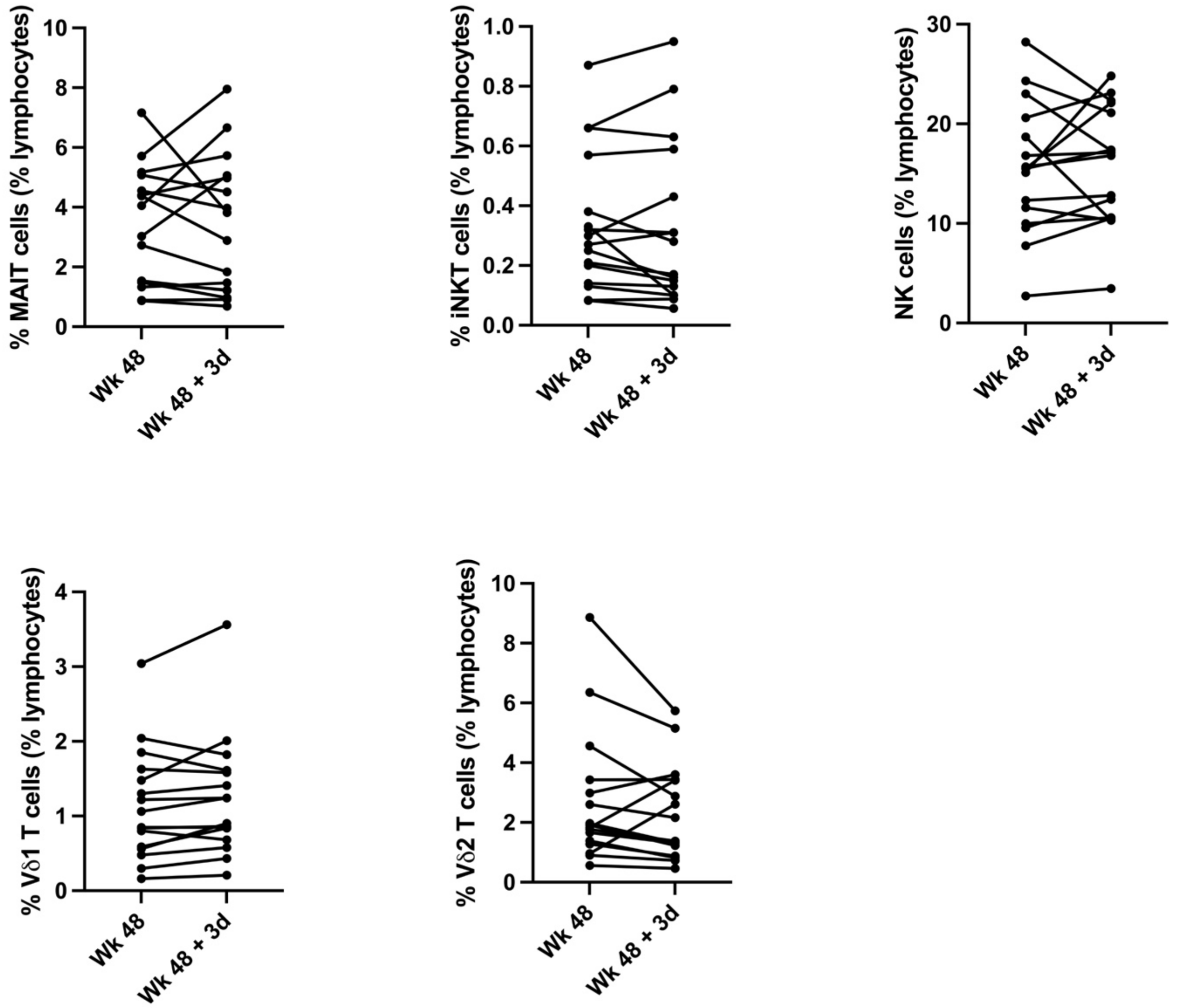

Supplemental Figure 3: Innate cell frequencies 3 days post vaccination. MAIT, iNKT, NK and Vδ T cell frequencies pre- and post-vaccination. Groups 2 (ALVAC-HIV + AIDSVAX B/E) and 3 (AIDSVAX B/E) are combined for analysis. (N=16).

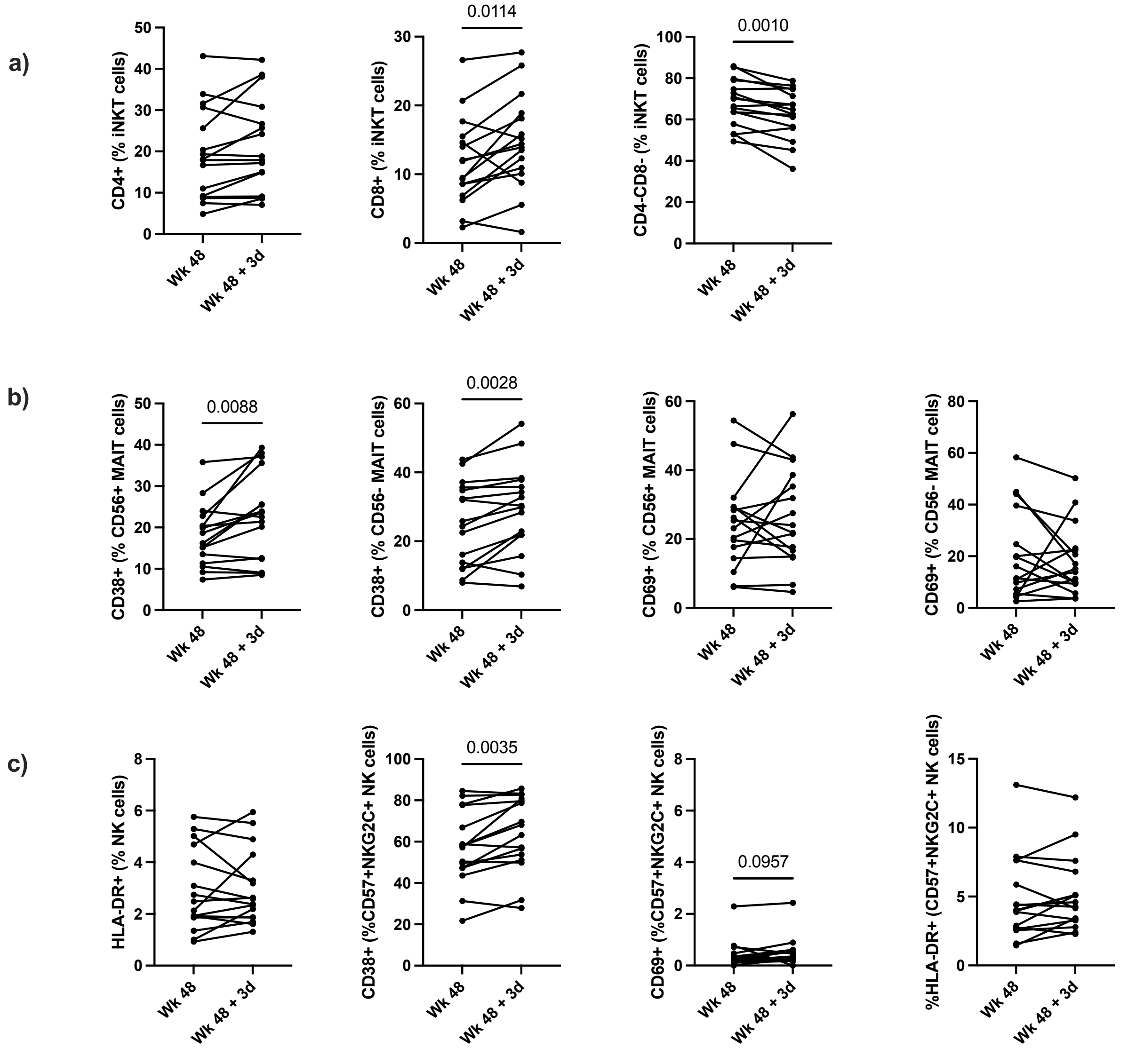

Supplemental Figure 4: Innate cell subset activation 3 days post vaccination. a) iNKT, b) MAIT, and c) NK cell subset activation pre- and post-vaccination. Groups 2 (ALVAC-HIV + AIDSVAX B/E) and 3 (AIDSVAX B/E) are combined for analysis. (N=16).

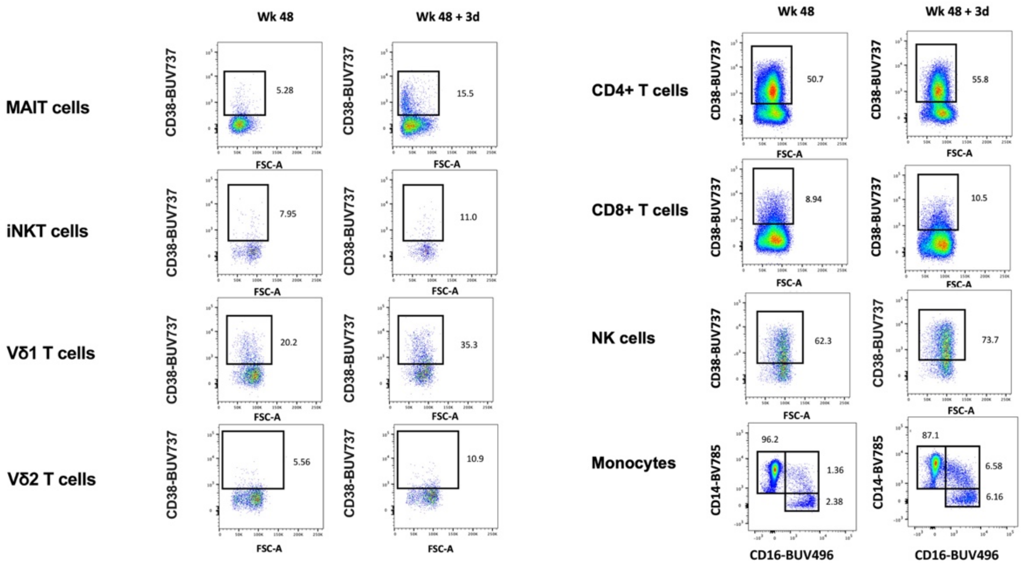

Supplemental Figure 5: Representative flow plots for innate cell and conventional CD4+ and CD8+ T cells activation pre- and post-vaccination and changes in monocyte frequency.

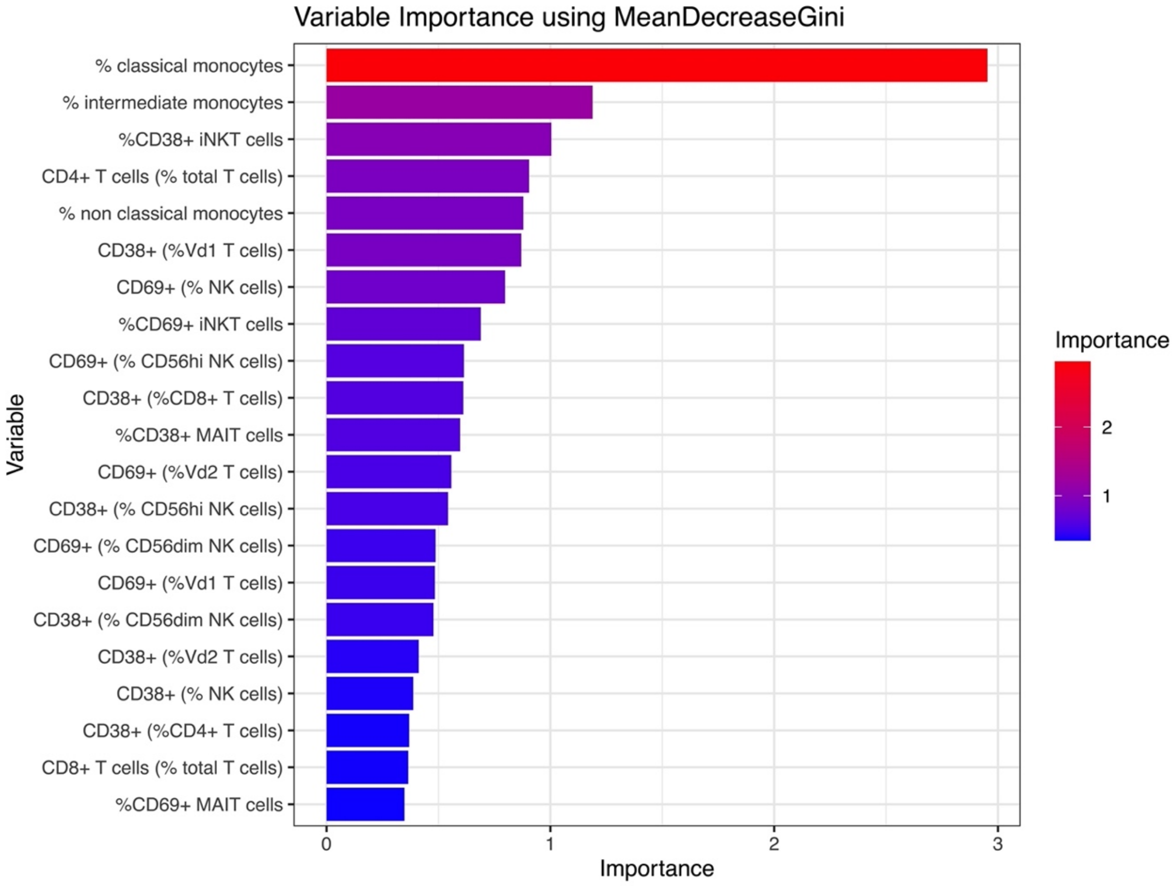

Supplemental Figure 6: Random Forest classification algorithm analysis. Hierarchical significance of immune markers as determined by their Gini Coefficient reductions within a random forest analysis. Immune cell types and their activation levels are displayed along the y-axis, with the x-axis representing their weighted impact on the predictive precision of the model through Gini Coefficient reductions. Groups 2 (ALVAC-HIV + AIDSVAX B/E) and 3 (AIDSVAX B/E) are combined for analysis. (N=16).

References

1. Pitisuttithum, P., et al., *Late boosting of the RV144 regimen with AIDSVAX B/E and ALVAC-HIV in HIV-uninfected Thai volunteers: a double-blind, randomised controlled trial.* Lancet HIV, 2020. **7**(4): p. e238-e248.
2. Shubin, Z., et al., *Additional boosting to the RV144 vaccine regimen increased Fc-mediated effector function magnitude but not durability.* AIDS, 2023. **37**(10): p. 1519-1524.
